# Characterization and Evaluation of Department of Veterans Affairs Commission on Accreditation of Rehabilitation Facilities–Accredited Interdisciplinary Pain Rehabilitation Programs: Protocol for a Mixed Methods Program Evaluation

**DOI:** 10.2196/72091

**Published:** 2025-05-05

**Authors:** Jolie N Haun, Christopher A Fowler, Dustin D French, Megan C McHugh, Jacquelyn N Heuer, Lisa M Ballistrea, Rachel C Benzinger, S Angel Klanchar, Friedhelm Sandbrink, Jennifer L Murphy

**Affiliations:** 1 Research and Development Service James A. Haley Veterans' Hospital Tampa, FL United States; 2 Division of Epidemiology Department of Internal Medicine University of Utah Salt Lake City, UT United States; 3 Department of Psychiatry and Behavioral Neurosciences University of South Florida Tampa, FL United States; 4 Health Services Research and Development Center of Innovation for Complex Chronic Healthcare Department of Veterans Affairs Edward Hines, Jr. VA Hospital Hines, IL United States; 5 Center for Health Services and Outcomes Research Feinberg School of Medicine Northwestern University Chicago, IL United States; 6 Departments of Ophthalmology and Medical Social Sciences Feinberg School of Medicine Northwestern University Chicago, IL United States; 7 Informatics, Decision-Enhancement and Analytic Sciences Center VA Salt Lake City Health Care System Salt Lake City, UT United States; 8 National Pain Management, Opioid Safety, and Prescription Drug Monitoring Program Specialty Care Program Office Veterans Health Administration Washington, DC United States; 9 Department of Neurology George Washington University Washington, DC United States

**Keywords:** chronic pain, veterans, interdisciplinary, pain program, pain rehabilitation, pain management, multidisciplinary

## Abstract

**Background:**

Veterans are more likely to experience chronic pain than civilians, with significant negative impacts on long-term health outcomes. Evidence for the effectiveness of prescription opioids for chronic pain management is limited, and chronic use of opioids is associated with an increased risk of sleep-disordered breathing, cardiovascular complications, and bowel dysfunction, as well as opioid misuse and overdose. Veterans Affairs (VA) and Department of Defense guidelines are prioritizing low-risk, evidence-based interdisciplinary pain management strategies while optimizing pain-related outcomes (PRO) for veterans. Commission on Accreditation of Rehabilitation Facilities (CARF)–Accredited VA Interdisciplinary Pain Rehabilitation Programs (IPRPs) have shared characteristics, while maintaining their unique characteristics as individual pain management programs. Though little is known about the characteristics of VA’s IPRPs (eg, staffing, services, and patients served), implementation, and sustainability of these mandated programs.

**Objective:**

The goals of our operational partner-driven evaluation are to (1) characterize IPRPs across multiple program factors, including but not limited to, service delivery methods, team composition, program characteristics, services and modalities offered, and patients served; (2) triangulate data to inform data visualization that characterizes and illustrates the IPRPs individually and collectively as a system of care; and (3) identify patient-reported outcomes (PROs) and metrics to measure program effectiveness and determine overlap across IPRPs.

**Methods:**

This partnered-driven program evaluation will use a sequential mixed methods prospective design, including interviews and surveys. The Consolidated Framework for Implementation Research (CFIR), Reach, Effectiveness, Adoption, Implementation, and Maintenance (RE-AIM) framework, and Expert Recommendations for Implementing Change (ERIC) strategies will be used to contextualize qualitative data. Rapid content analysis will be used to iteratively analyze qualitative data, while descriptive statistics will be used to analyze quantitative data. Datasets will be triangulated to support data visualization for partners to inform clinical and operational decision support.

**Results:**

As of April 2025, All IPRP sites are engaged, and survey and interview data have been collected and prepared for analysis. The results and deliverables will inform VA CARF-accredited IPRP characterization, evaluation, and implementation as a learning health system.

**Conclusions:**

The results of this evaluation will characterize CARF-accredited IPRPs and identify determinants affecting the implementation of this complex intervention, made up of multiple evidence-based practices. Partner-driven data will inform the state of implementation at each site, and quantitative measures will provide options for collecting standardized outcome measures for continued program evaluation. This operational partner-driven evaluation will inform future efforts for quality improvement to improve veterans’ pain management outcomes. This protocol informs the use of a mixed methods approach to evaluate a multimodal intervention (ie, IPRP), made up of multiple evidence-based practices to treat a complex comorbid condition. Future work may include data management infrastructure development and cost evaluations to inform clinical and operational decision-making.

**International Registered Report Identifier (IRRID):**

DERR1-10.2196/72091

## Introduction

### Background

More than 50 million American adults (20.4%) are estimated to have chronic pain [1]. Chronic pain is defined as persistent pain lasting more than 3-6 months after initial onset [2]. High-impact chronic pain is indicated by the presence of pain that significantly reduces social, occupational, and self-care activities on at least half of the days over this time period [3,4]. Compared with civilians, veterans are more likely to experience chronic pain (29.1% vs 19.5%) [1] and high-impact chronic pain (9.1% vs 6.4%), with significant negative impacts on long-term quality of life [5] and affecting overall functioning [6,7]. In an effort to manage chronic pain, some veterans become reliant on prescription opioids [8,9], which have numerous unintended health consequences such as increased risk of sleep-disordered breathing, cardiovascular complication, and bowel dysfunction, as well as opioid misuse and overdose [10-13]. In recent years, there has been increased attention on low-risk interdisciplinary treatment options for chronic pain management in the Department of Veterans Affairs (VA) system [14].

Currently, VA promotes an interdisciplinary integrative approach to pain management [15,16]. Subtitle A of the 2016 Comprehensive Addiction and Recovery Act (CARA) mandates that each VA medical center have a designated pain management team to treat and coordinate pain care for veterans [15]. This approach to pain management favors a biopsychosocial approach, which conceptualizes chronic pain conditions as complex and multidimensional, emphasizing interdisciplinary integrated models of care, as outlined in the VA’s Stepped Care Model for Pain Management (SCM-PM) [16-18]. Current VA and Department of Defense guidelines suggest that the potential benefits of interdisciplinary pain treatment for complex comorbid conditions including substance use disorders, outweigh potential harms [16]. The VA’s Interdisciplinary Pain Rehabilitation Programs (IPRPs) are empirically supported for improving pain-related outcomes (PRO) in the veteran population [14]. Policy [17,18] and evidence-based practice [14,19] have fueled the expansion of the VA’s IPRPs. Despite implementation throughout the system, little is known about the characteristics of the IPRPs (eg, staffing, services, and patients served), including implementation and sustainability factors (Office of Pain Management, Opioid Safety, and Prescription Drug Monitoring Program [PMOP], personal communication, March 2023).

This protocol describes a national evaluation designed to characterize the VA Step 3 Commission on Accreditation for Rehabilitation Facilities (CARF)–accredited IPRPs. The aims of this project were to conduct an evaluation of IPRPs to (1) characterize IPRPs across multiple program factors, including but not limited to, service delivery methods, team composition, program characteristics, services and modalities offered, and patients served; (2) triangulate data to inform data visualization to characterize and illustrate the IPRPs individually and collectively as a system of care; and (3) identify patient-reported outcomes (PROs) and metrics to measure program effectiveness and determine overlap across IPRPs. Deliverables from this project will support IPRP implementation and sustainability. Protocol components are reported in accordance with the SQUIRE 2.0 (Standards for QUality Improvement Reporting Excellence) guidelines [20].

### IPRP Overview

Published in 2009, Veterans Health Administration (VHA) Directive 2009-053 [18] established the SCM-PM as a practical framework to contextualize the national standards of care for treating veterans with chronic pain based on needs and risk. The SCM-PM begins with low-intensity interventions delivered in primary care settings where more common pain conditions are seen (step 1) and progresses to specialty pain care services provided by interdisciplinary pain management teams (step 2), and step 3 tertiary pain care based on pain complexity, risk factors, and veteran needs (eg, comorbidities), which includes services such as IPRPs [17]. In addition, VHA Directive 2009-053 mandated the implementation of at least one CARF-accredited IPRP in each region of the VHA (ie, Veterans Integrated Service Networks [VISNs]) [18]. In the 10 years following the publication of VHA Directive 2009-053, the number of CARF-accredited IPRPs in VHA increased 10-fold, from 2 to 20. Conversely, CARF-accredited IPRPs in the US (outside the VA) decreased from 210 in 1998 to 87 in 2019 (58.57%) [14,19]. Since 2019, additional VA IPRPs have continued to pursue and receive CARF-accreditation. VA also has a robust community of practice (CoP) for IPRPs to share information and support. Important congressional mandates including CARA [15], and the emergence of the national VA whole health-oriented, integrative health care priorities [21] reinforce the importance of IPRP development and implementation.

Interdisciplinary pain rehabilitation programs consist of multimodal, interdisciplinary provider teams with a shared “mission, philosophy, and set of objectives” to improve patient care using a biopsychosocial approach [7]. By design, IPRPs support interdependence among team members, which allows for consistent, open communication and collaboration with decision-making, as well as setting, reviewing, and revising treatment goals [7,19,22,23]. This integrated team-based approach is conducive to providing evidence-based, person-centered care that addresses the multifactorial elements of chronic pain.

Interdisciplinary pain rehabilitation programs may be heterogenous with respect to numerous characteristics (eg, team composition, modalities offered, program length, etc) [7,14,19,23-25]. The most common team members include medical (ie, physicians, nurse practitioners, and physicians’ assistants), behavioral health (ie, psychologists and social workers), and rehabilitation (ie, physical therapists and occupational therapists), with the expected associated modalities provided, respectively (ie, medical management, behavioral therapies, and exercise programs). Less represented but highly valuable additional team members may include pharmacists, nutritionists and dietitians, recreation therapists, vocational rehabilitation specialists, support staff, health and wellness coaches, nurses, nurse program coordinators, and administrative support [7,14,23]. Team integration, regular communication, and care coordination are shared philosophies of IPRPs [7,14,19,22]. More comprehensive overviews of IPRP are published elsewhere [7,19,22,26,27].

### Empirical Support for IPRPs

Systematic reviews and meta-analyses support the effectiveness of IPRPs with most evidence coming from the public sector. Evidence from a focused review and meta-analysis of interdisciplinary pain rehabilitation pre-post and follow-up effects [25] found pre-post improvement across a variety of pain-related (intensity and interference), psychological (anger, anxiety, depression, and self-efficacy), and functional (physical and social) PROs, with improvements typically maintained at follow-up. A review of 4 model IPRPs largely supported these findings across similar outcomes [23]. Compared with usual care, IPRPs have demonstrated small size improvements for pain intensity and disability, with moderate evidence quality [28]. Meta-analyses comparing IPRPs to active interventions (eg, physical treatments and surgery) found medium effect sizes for improvements in pain and disability but with significant heterogeneity in findings and low evidence quality [28,29].

The VA is a national leader in IPRP delivery and growth with various veteran outcomes studies. Murphy et al [30,31] examined large national samples of veterans that completed the CARF-accredited Tampa VA inpatient IPRP. Veterans typically experienced pre-post improvements in pain-related (intensity, interference, fear of movement, and catastrophizing), psychological (negative affect and relaxation), and functional (vitality and sleep) PROs with effect sizes ranging from small to large. Outcomes were similar between veterans who tapered off of their prescription opioids during the program compared with those not taking opioids [30]. More recent small-scale studies at the CARF-accredited San Francisco VA IPRP found that veterans experienced pre-post improvements in pain catastrophizing, depression, as well as disability, with mixed results for pain intensity [32,33]. A recent study by this group found mostly similar results between their in-person (prepandemic) and online (postpandemic) program iterations with lower dropout in the online program [34]. Finally, a large multisite VA IPRP evaluation examined pre-post improvements on common PRO measures administered across all sites. They also characterized each IPRP (Albuquerque, New Mexico; Cleveland, Ohio; Puget Sound, Washington; San Francisco, California; Tampa, Florida [inpatient], Tampa, Florida [outpatient]) including factors such as program time commitment, team composition, and modalities offered. This study found that VA IPRPs were heterogenous in their program structures. Significant improvements in pain-related outcomes including intensity, catastrophizing, interference (mobility and activities of daily living), fear of movement, negative affect, vitality, and insomnia severity were observed. Aggregate effect sizes across IPRPs were in the small-to-medium range.

Interdisciplinary pain rehabilitation programs are an essential part of complex pain care and are empirically supported for improving PROs in the veteran population [14]. There has been important policy support [17,18] and substantial clinician-led grassroots efforts to expand IPRPs in the VA system [14,19]. This national evaluation to characterize the VA step 3 CARF-accredited IPRPs will support current and future implementation, sustainability, and policy for these valued programs. This protocol leverages established methods for evaluating multimodal interventions made up of multiple evidence-based practices for complex comorbid health conditions [35]. This approach can be applied to evaluating pain rehabilitation in VA and non-VA learning health systems in high-reliability organizations.

## Methods

### Concept and Design

**Figure 1:**
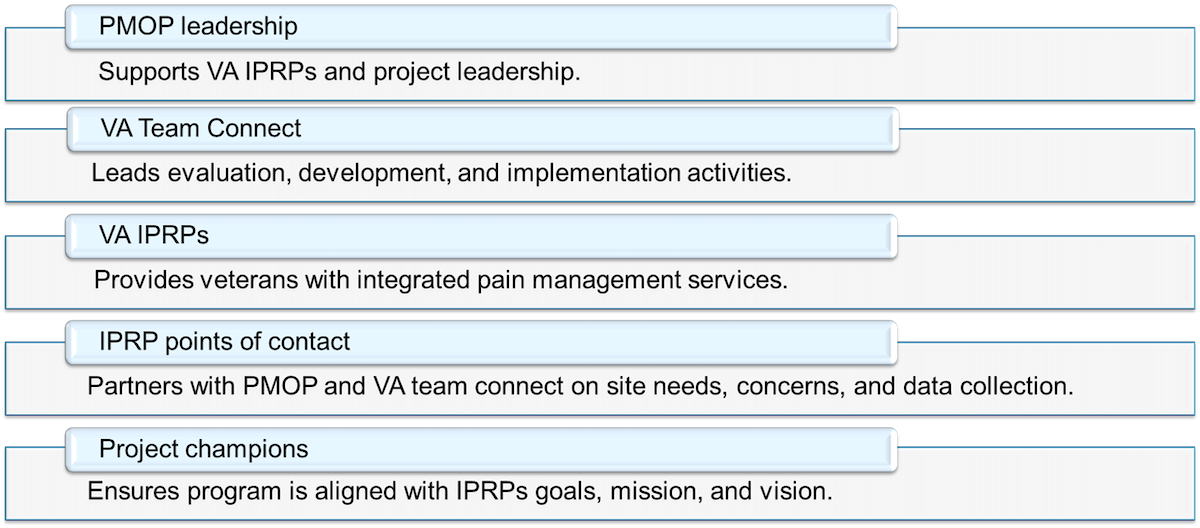
Interdisciplinary Pain Rehabilitation Programs (IPRPs) partnered evaluation collaborative roles. PMOP: Department of Veterans Affairs Office of Pain Management, Opioid Safety, and Prescription Drug Monitoring Program. VA: Veterans Affairs. IPRP: Interdisciplinary Pain Rehabilitation Program.

This partnered evaluation will be conducted through a sequential mixed methods process [36,37] and in close collaboration with the study sponsors. Our team has established methods for evaluating complex interventions made up of multiple evidence-based practices for complex comorbid health conditions such as chronic pain [35]. In this partnered mixed methods sequential design, we will use multiple steps. In step 1, our operational partners will provide us with the list of VA step 3 CARF-accredited IPRPs to be evaluated including points of contact (POCs) to be recruited for key informant interviews. In step 2, POCs that will be targeted for recruitment will be able to refer additional key informants for recruitment. In step 3, we will leverage the previous characterization of a subset of VA step 3 CARF-accredited IPRPs [14] as the starting point for the development of this protocol. In step 4, we will iteratively develop the qualitative interview guides and program characteristics survey through multiple rounds of partnered feedback. In step 5, following data collection and analysis, we will deliver findings to our partners who validate our data and iteratively advise us on whether additional information needs to be collected. Finally, in step 6, we will leverage relationships with partners at the operations and IPRP levels to determine dissemination opportunities and product development (Figure 1).

### Sampling and Sample Size

This program evaluation will use purposive and referral-based sampling methodologies to recruit participants for key informant interviews at the population level for all CARF-accredited and accreditation-seeking VA IPRPs. Specifically, a planning meeting between the research team and the study sponsors was held in July 2023 to clarify and discuss the goals of the evaluation. The sponsors provided the evaluation team with names and contact information for POCs at all CARF-accredited and accreditation-seeking VA IPRPs. These POCs will be targeted as key informants to provide information about their IPRPs. Furthermore, POCs from each IPRP, typically the program director, will also be able to identify and refer additional IPRP personnel (eg, former IPRP director, data manager, program coordinator, etc) to be recruited for key informant interviews.

**Figure 2:**
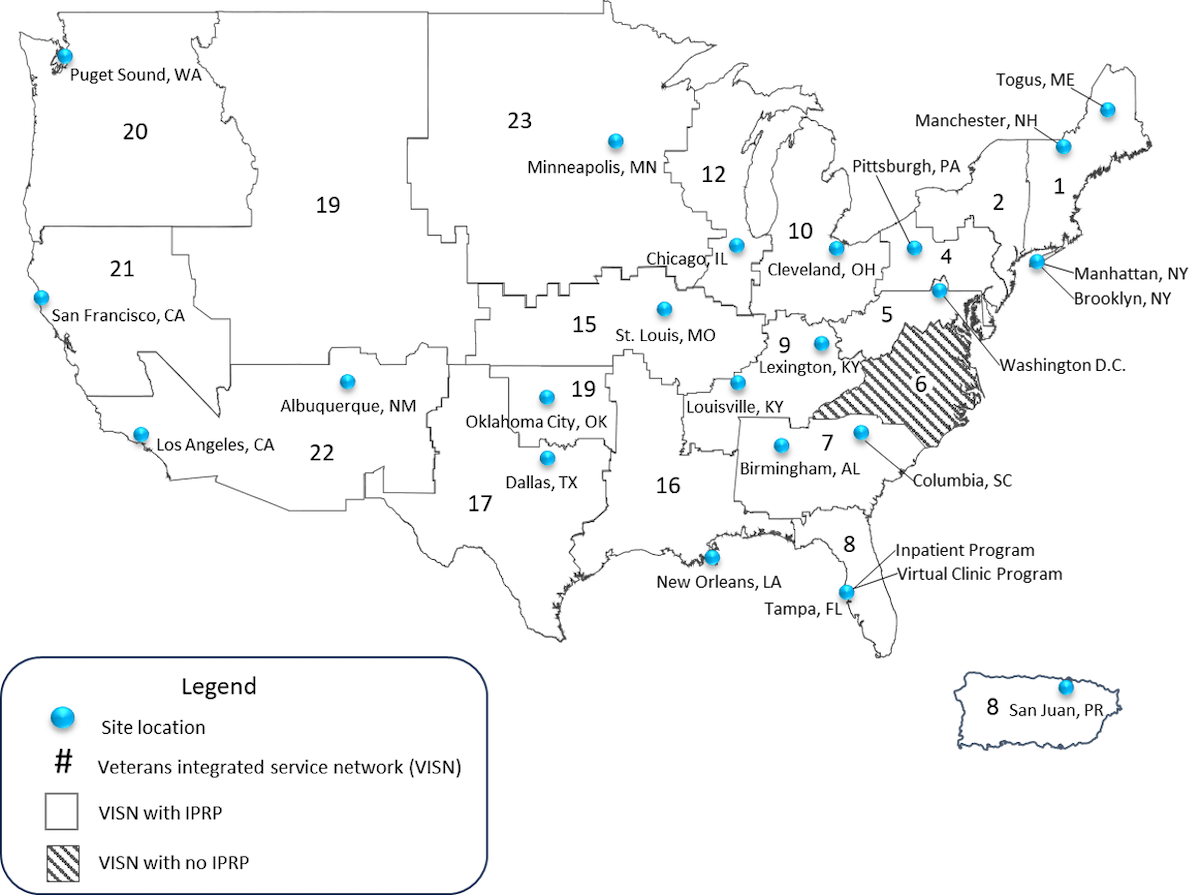
Pain Management, Opioid Safety, and Prescription Drug Monitoring Program (PMOP) in Interdisciplinary Pain Rehabilitation Programs (IPRPs) site map. IPRP: Interdisciplinary Pain Rehabilitation Program.

In total, 23 VA IPRPS (21 CARF accredited and 2 in the accreditation process) will be evaluated (Figure 2). Furthermore, one of the CARF-accredited VA IPRPs is mirrored across two independent VA medical centers (Brooklyn and Manhattan). We will conduct separate interviews with a POC at each of these VA sites which have their own independent staffing and within-facility factors (eg, collaboration, team dynamics, community resources, etc). In total, 24 POCs will be targeted to complete key informant interviews and an IPRP characteristics survey.

### Qualitative Interview Guides

Two semistructured key informant interview guides will be iteratively developed with operational partners. The first interview guide will contain broad questions about the strengths and weaknesses of the IPRPs, how the IPRP coordinates with other pain services within and outside the facility, changes to the program over time, and the use of telehealth. The second interview guide will emphasize data collection, analysis, and use of PRO measures, as well as collaboration with other IPRPs. Interview guides will be structured by the domains of implementation, as outlined by the Consolidated Framework for Implementation Research (CFIR) [38]. Interview guides were piloted internally by the qualitative team to ensure flow and proper timing.

### Program Characteristics Survey

The IPRP program characteristics survey will target specific information about the size and scope of VA IPRPs. This survey was designed based on characteristics from a previous IPRP multisite evaluation [14] and input from our operational partners. These will include the number of veterans served, treatment modalities, staffing, and service delivery among other characteristics (refer to Table 1).

**Table 1:** Interdisciplinary Pain Rehabilitation Program (IPRP) characteristics.

Characteristic and scale			Description
**IPRP^a^ time commitment**			
	Program length (weeks)	Total program time commitment for veterans participating in IPRP.	
	Days per week	—^b^	
	Hours per day	—	
	Total hours	—	
**Mode of delivery**			
	In-person (Y or N^c^)	IPRP offers in-person or online health delivery of their services.	
	Online (Y or N)	—	
**Team composition**			
	Specialty (Y or N)	Team members’ specialties and time commitment dedicated to delivering IPRP services.	
	FTE^d^ (%)	—	
**Services offered**			
	Services (Y or N)	Treatment modalities offered by IPRPs (eg, physical therapy, behavioral therapies, medication management, etc).	
**Collaboration**			
	Collaboration (Y or N)	Services that IPRPs collaborate with (eg, services, referrals, resource-sharing, etc).	
**IPRP referrals, enrollment, and capacity**			
	Referrals	Total number of veterans referred to IPRP in FY^e^23.	
	Acceptance rate	Total number of veterans accepted to IPRP in FY23.	
	Enrollment	Total number of veterans enrolled in IPRP in FY23.	
	Program completers	Total number of veterans that completed IPRP in FY23.	
**Program outcomes**			
	Metrics	PROs^f^ and metrics used to assess IPRP effectiveness.	

^a^IPRP: Interdisciplinary Pain Rehabilitation Program.

^b^Not applicable.

^c^Y or N: yes or no.

^d^FTE: full-time employment.

^e^FY: fiscal year.

^f^PROs: patient-reported outcomes.

### Procedure

This evaluation will be promoted to IPRPs using multiple approaches. In October 2023, the study author (CAF) informally presented the project and recruitment process at the monthly VA IPRP CoP call. In January 2024, author CAF provided a 45-minute “site activation” presentation to describe the evaluation and expectations on the VA IPRP CoP call. The study sponsor (PMOP) distributed a national memo to describe the evaluation and anticipated activities with input from study authors (CAF, JNH, and LMB). From March to May 2024, authors (CAF and JNH) attended and presented the evaluation at 3 monthly VA regional pain conferences.

IPRP POCs will be contacted by a study team member (SAK) via email to schedule their interview. Key informant interviews will be led by one of three experienced qualitative researchers (MCM, and JNH) and a second research team member will take notes during the interview (JNH, BH, CAF, and LMB).

Following the completion of their first interview, POCs will be sent the program characteristics survey via Qualtrics (Qualtrics International Inc), a Federal Risk and Authorization Management Program that is an approved, secure, cloud-based data collection platform [39] that has demonstrated feasibility for electronic data collection within the VA system [40]. If POCs do not complete the survey before the second interview, the interviewer will complete it with the respondent during the interview. A team member (CAF) will serve as the IPRP liaison and follow up with IPRP POCs for clarifying information as needed. The follow-up key informant interview will be scheduled and completed using the same procedures as the first interview. The final interview protocols and survey instrument can be found in Multimedia Appendix 1.

### Qualitative Analysis

Interview data will be analyzed using rapid iterative qualitative analysis [41]. The process will begin with the generation of field notes by the note taker, which will then be reviewed and verified by the interviewer. The field notes will then be exported into a Microsoft Excel (Microsoft Corp) workbook for rapid coding and analysis. The three interviewers will develop a coding scheme consisting of deductive, relevant codes applicable to questions about program facilitators, barriers, challenges, and strengths. In addition, they will develop an inductive coding scheme for questions involving specific program issues (eg, Who enters the PRO data and where is the data entered?). The inductive coding scheme is designed to group participant responses. The 3 team members will code one interview together, and then 2 interviews individually to compare responses. This process will lead to some refining of the coding schemes. Next, the 3 team members will independently code an additional interview, and intercoder reliability will be established. All remaining interviews will be coded by a single team member and then verified by a second team member.

Once coding is completed, the codes for individual questions will be totaled, and team members will develop brief descriptions of the trends and findings from the coded data. These descriptions will then be thematically sorted using CFIR [42], Reach, Effectiveness, Adoption, Implementation, Maintenance (RE-AIM) [43], Levesque’s model of access [44], and Powell’s implementation strategies [45]. These themes will be used to organize the findings, which will then be organized into descriptive and comparison matrices to summarize the findings and highlight key differences between sites. When applicable, specific examples and salient quotes will be pulled from the field notes to help illustrate key findings. During this process of data collection and analysis, the research team will meet regularly to discuss emerging findings. Data will be extracted and analyzed to characterize VA CARF-accredited IPRPs and to develop an implementation research logic model [46,47]. In addition to the team’s process of validating the reliability of the analysis, the team will iteratively engage IPRP representatives and operational partners to address the validity, dependability, and representation of data findings and summaries. This partnered approach ensures data validation and representation from the partners’ perspective and data outcomes [48,49].

### Quantitative Analysis

Survey data will be analyzed using basic descriptive statistics (eg, counts and means) to display data in tables and figures. For participant demographics and IPRP characteristics, frequencies and percentages will be used to describe categorical variables. Normally distributed continuous variables will be described using their range, mean, and SD. Skewed continuous variables will be described using range, median, and IQR.

### Triangulation of Data

Qualitative and quantitative datasets will be triangulated to contextualize the characterization of the IPRPs, as well as understand the determinants, strategies, and outcomes associated with their implementation, per our conceptual model. When appropriate, programmatic quantitative descriptive data may be used to create cohorts of IPRPs that share characteristics to explore and understand qualitative findings. Likewise, qualitative findings on determinants and strategies may be triangulated with quantitative data to examine relationships reflecting the sites’ unique characteristics.

### Ethical Considerations

The project protocol was reviewed by the Research and Development Committee of the James A. Haley Veterans’ Hospital (IRBNet 1777324). This project was determined to be nonresearch, and Institutional Review Board approval was not required because the data collection activities were for quality improvement and not for research. Participants only provided consent to be recorded during interviews for data confidentiality purposes. All methods were carried out in accordance with relevant guidelines and regulations. All staff interview tools, including interview guides and the program characteristics survey, were approved by the National VA Union. No compensation was provided to the participants in this quality improvement project. Participants’ identities were confidential and deidentified, and data were stored behind the VA firewall. Data will be presented anonymously in an aggregate form in accordance with the VHA policies and regulations. Nevertheless, all participants will be informed of the purpose and intended use of the data collected, assured confidentiality, and informed that they can decline to answer any question or cease participation at any point without penalty. All interviews will be completed via videoconference using Microsoft Teams (Microsoft Corp). All respondents will be provided oral consent to participate in the interviews.

## Results

Project activities and funding began in October 2023. As of April 2025, key informant interviews have been completed at all 23 IPRPs. The first key informant interviews (n=31) were completed between February and May 2024. Follow-up key informant interviews (n=30) were completed between May and July 2024. Program characteristics surveys have been completed by 21 of 23 IPRPs and will be finalized for the remaining two sites. Findings will be disseminated in mid-2025 and thereafter to inform characterization and support program-level and system-level efforts.

## Discussion

### Principal Findings

The primary goal of this evaluation protocol is to complete the first system-wide characterization of VA step 3 IPRPs to better understand these unique programs. Findings from this mixed methods evaluation will identify targets for supporting VA IPRPs through multiple mechanisms. First, findings will inform the development of a measure-outcomes infrastructure to provide IPRPs with better access to system-wide IPRP data based on an adopted PMOP Pain Measures Set. This process will also establish a capacity to conduct standardized evaluations of the effectiveness of VA IPRPs from the individual program level to the system-level [50]. Second, the findings will provide a framework for further characterization, implementation, and evaluation efforts across the VA IPRP system. Third, this evaluation will inform system-wide PMOP funding and staffing initiatives directed toward facilitating and sustaining the adoption of state-of-the-art pain management practices within the VA. These steps are essential to support PMOP and step 3 IPRPs as a learning health system.

### Future Directions

This protocol is the first phase of a 5-year VA IPRP evaluation and implementation project. In fiscal year (FY) 2025, our team will begin phase 2 with the primary focus on the iterative development of a dynamic data dashboard with the capacity to collect and descriptively analyze pain outcomes from a recently implemented PMOP Pain Measures Set [50], as well as other key metrics (eg, Veterans served). The PMOP Pain Measures Set is a brief multidimensional PRO battery designed to introduce a standardized measurement plan to assess VA pain management services nationwide including pain intensity, pain interference, pain catastrophizing, pain self-efficacy, anxiety, depression, sleep quality, subjective health status, overall well-being, and perceived impact of treatment. The first iteration of this dashboard will be informed by barriers and facilitators to data collection and analysis reported by key informants during qualitative interviews. Primary goals for dashboard development are to (1) streamline data collection, analysis, and reporting to reduce the burden for the individual IPRPs, (2) encourage communication and collaboration among IPRPs to learn from each other on how to improve their outcomes, and (3) to build capacity to conduct regular evaluations of the IPRPs across the VA system. Human-centered design and partner feedback will be used throughout the iterative development of the IPRP dashboard. The latter goal fulfills the VHA Directive 2009-053 mandate for IPRPs to conduct consistent and timely assessments of veterans’ pain outcomes to examine the effectiveness of pain management interventions [18].

Phase 3 (FY26) of this evaluation will conduct mixed methods efforts including piloting our dynamic data dashboard to collect PMOP Pain Measures Set [50] outcomes data in a small subsample of champion VA IPRPs. Our data dashboard will be used to provide dynamic reporting of descriptive PROs and additional program statistics collected by our dashboard to our IPRP partners. This quantitative data collection effort will be used to determine the feasibility and initial efficacy of our data dashboard. Key informant interviews conducted at pilot sites will inform a second iteration of our data dashboard to meet current and future PMOP and VA IPRP needs.

In the final phase (FY27-28), we will expand on our pilot study by implementing our dynamic data dashboard to all CARF-accredited VA IPRPs and conduct a mixed methods 12-month national evaluation. An expanded version of the PMOP Pain Measures Set will be implemented in the future, which will allow for more consistent evaluation of PROs across programs. The collection of additional data will allow us to estimate the number of veterans served by all VA IPRPs in real time. Key informant interviews will be conducted to gain insights into the usability of our centralized data collection dashboard system-wide. Qualitative data will also be leveraged to continue iteratively tailoring our data dashboard to meet the future needs of our IPRP partners.

### Limitations

There are multiple limitations to consider when reviewing this protocol. First, at the current time, we do not have a methodology for capturing real-time changes in IPRP staffing or implementation of new modalities, which can address the needs of these programs in a timely manner. While this is out of the scope of the current project, a staffing tool is being piloted to address pain program staffing needs across the VA [51]. A second potential limitation is aiming for 0% attrition across 23 IPRPs for 3 study activities (eg, initial interview, program characteristics survey, and follow-up interview). Several steps are taken to help facilitate participation including (1) multiple presentations to the target audience to present the evaluation aims and potential benefits to IPRP, and (2) drafting a memo with PMOP to outline anticipated program participation. Third, POCs may have misconceptions regarding the purpose of the interview and provide socially desirable or expected responses (eg, demand characteristics) [52]. The evaluation team will aim to reduce these effects by providing transparency throughout the process, including informing participants that their interview responses will be examined in the aggregate, their names will not be linked to any of their responses, and that this evaluation is designed to develop mechanisms to improve the IPRP process. Finally, the current evaluation does not address the cost-effectiveness of VA IPRPs, which is beyond the scope of this project. However, this remains an important area of future study because IPRPs are very resource-intensive but have demonstrated cost-effectiveness in the private sector via reduced service utilization postrehabilitation [22,26].

### Management Plan and Timeline

Operational partners were engaged to develop a comprehensive timeline for anticipated deliverables (Multimedia Appendix 2). Their input also informed the development of the proposed work and approval of evaluation methods and processes. In addition to project deliverables, the evaluation team will develop quarterly and annual reports to keep partners abreast of study progress (eg, current and completed tasks, recruitment, data collection, preliminary findings, and lessons learned). Our partners will also provide feedback on deliverables (eg, presentations and papers) and tools developed for the current evaluation (eg, interview guides and program characteristics survey).

### Conclusions

In summary, the characterization of the VA’s CARF-accredited pain programs is critical to the national-level program evaluation of outcomes. This effort is also informing the development of a national effort to implement PRO measurement infrastructure for quality improvement to optimally serve veterans in managing their chronic pain. These data will support efforts to establish CARF-accredited pain programs as part of the VA learning health system. The successful implementation and dissemination of this program evaluation will contribute to (1) optimizing pain management services for veterans, (2) developing an infrastructure to monitor patient outcomes regarding health and function, (3) advancing the science of evaluating complex interventions (ie, multiple evidence-based practices) for complex comorbid conditions associated with pain management, and (4) demonstration of VA CARF-accredited pain programs as a learning health system.

## Appendix

Multimedia Appendix 1 Interview protocols and survey instrument.

Multimedia Appendix 2 Timeline of study activities by year.

## Abbreviations

CARA: Comprehensive Addiction and Recovery Act
CARF: Commission on Accreditation of Rehabilitation Facilities
CFIR: Consolidated Framework for Implementation Research
CoP: community of practice
FY: fiscal year
IPRP: Interdisciplinary Pain Rehabilitation Program
PMOP: Pain Management, Opioid Safety, and Prescription Drug Monitoring Program
POC: point of contact
PRO: patient-reported outcome
RE-AIM: Reach, Effectiveness, Adoption, Implementation, and Maintenance
SCM-PM: Stepped Care Model for Pain Management
SQUIRE 2.0: Standards for QUality Improvement Reporting Excellence
VA: Department of Veterans Affairs
VHA: Veterans Health Administration

## Data Availability

Data for this project are available from the authors upon reasonable request. The corresponding author, as a US government employee, has the right to grant on behalf of all authors and hereby does grant on behalf of all authors, a nonexclusive worldwide license to the publishers and its licensees in perpetuity, in all forms, formats, and media (whether known now or created in the future), to (1) publish, reproduce, distribute, display, and store the contribution; (2) translate the contribution into other languages, create adaptations, reprints, include it within collections, and create summaries, extracts, and/or abstracts of the contribution; (3) create any other derivative work(s) based on the contribution; (4) exploit all subsidiary rights in the contribution; (5) include electronic links from the contribution to third-party material, wherever it may be located; and (6) licensee any third party to do any or all of the above.
