# Supplementary material for: Characterization and Evaluation of Department of Veterans Affairs Commission on Accreditation of Rehabilitation Facilities–Accredited Interdisciplinary Pain Rehabilitation Programs: Protocol for a Mixed Methods Program Evaluation
Source: JMIR Res Protoc. 2025 May 5;14:e72091. doi: 10.2196/72091 (PMC12089867; doi:10.2196/72091)
Supplement: Multimedia Appendix 1 [file resprot_v14i1e72091_app1.docx]

Interview Guide – Interview #1

PMOP Project

Hello. My name is XXXX.

Thank you for agreeing to participate in this voluntary interview to help PMOP collect information about Step 3 interdisciplinary pain rehabilitation programs so that they can better support those programs. I have several questions for you about your program’s characteristics, collection of quality information, collaboration with other programs, and any operational challenges your program is experiencing.

If you do not know the answer to a question, I will ask you to identify someone else who I might contact to get the information.

I would like to audio-record this session to ensure accuracy in documenting your responses. However, your specific responses will not be linked to your name in our files and will remain confidential. The responses you provide will be aggregated with the responses attained from leaders of other VA interdisciplinary pain rehabilitation programs.

Do you have any questions before we begin? Do I have your permission to record this session?

**Respondent Information**

| Participant Name: | Job Title: |
| --- | --- |
| VAMC Facility: | Time at Facility: |
| Clinical Discipline: | Time w/ CARF Program: |
| Time at VA: | Time in Healthcare: |
| This cell collected information on identity of sex but has been omitted from this document post hoc to be in compliance with Executive Order 14168. | Race:  White/Caucasian  Black/African American  Asian  American Indian/Alaska Native  Unknown  Other (please list): __________  Decline to respond |
| Ethnicity:  Hispanic/Latinx  Not Hispanic/Latinx  Decline to respond | Highest Professional Degree Attained: |

**Interview #1**

**Program Background**

We are interested in learning more about your program.

1. Tell me more about your program. What are the main things you want us to know about your program?
2. What are your program’s strengths?
3. What are your program’s weaknesses?

**Program Environment and Coordination**

We are interested in understanding how your program is connected to, and part of, pain resources within your facility.

1. Is your program a stand-alone clinic or part of a larger clinic or program within your facility?
   1. *How does your program coordinate with other pain clinics or programs that exist within the facility?
   2. Is there an active management of pain (AMP) program at your facility? Are you working to establish one?
2. How would you describe the relationship between team members from your program and the team members from the other pain programs? (e.g., supportive, collaborative, competitive)?
3. How do team members across the programs coordinate services?
4. How do team members across the programs coordinate resources or information?
5. What characteristics of the broader VA facility help to support your program?
6. What characteristics present challenges?

**Enrollment [from PMOP]**

1. *How are Veterans recruited or referred into your program?
   1. *What are the primary sources of referrals?
   2. *How does your program track referrals and enrollment?
2. *What are the most common reasons why a referred Veteran may not be accepted into your program?
3. *Once referred, what are the most common reasons why a Veteran does not enroll in your program?
4. *What are the most common reasons why Veterans drop out or do not complete your program?

**Change over Time**

1. *How has enrollment in your program changed over time?
2. *How has the scope of services provided changed over time?
3. *How has the team composition changed over time?
4. *How did the COVID-19 pandemic impact referrals and programming?
5. Do you offer telehealth services? How are telehealth services used?
   1. If used, what factors facilitated the adoption of telehealth services?
   2. If not used, what factors are barriers to the adoption of telehealth services?

**Closing and Reminder**

Thank you for your time and participation in this interview. During interview #2, we will focus on understanding how the pain and psychological health outcome measures from the program characteristics worksheet are collected and how frequently they are collected. This will help us to better understand whether and how your program is connected to other sites.

If you haven’t done so already, please be sure to complete the Program Characteristics Worksheet before our next interview.

Interview Guide – Interview #2

PMOP Project

Hello. My name is XXXX.

Thank you for agreeing to participate in this voluntary interview to help PMOP collect information about Step 3 interdisciplinary pain rehabilitation programs so that they can better support those programs. I have several questions for you about your program’s collection of quality information and collaboration with other programs. Like other Team Connect national projects, we have been charged with understanding what your experiences are so that we can accurately reflect your program and your needs.

If you do not know the answer to a question, I will ask you to identify someone else who I might contact to get the information.

I would like to audio-record this session to ensure accuracy in documenting your responses. However, your specific responses will not be linked to your name in our files so your specific responses will not be identifiable. The responses you provide will be aggregated with the responses from leaders of other VA interdisciplinary pain rehabilitation programs.

Do you have any questions before we begin? Do I have your permission to record this session?

*[Begin recording.]*

Now that we’ve begun the recording, could you please confirm your consent to the recording for us?

**Interview #2**

**Program Characteristics Worksheet**

*[If respondent completed worksheet in advance of the call and all information is complete.]*

Thank you for taking the time to complete the program characteristics worksheet in advance of your call.

*[Proceed to next section.]*

*[If respondent completed worksheet in advance of the call and clarification is needed/items are missing.]*

Thank you for taking the time to complete the program characteristics worksheet in advance of your call. I would just like to clarify a few things…

*[If respondent did not complete the worksheet in advance of the call.]*

I would like to begin by completing the program characteristics worksheet with you…

***Qualtrics Link***

**Outcomes and Evaluation**

I would like to better understand how patient-reported outcome measures (e.g., PTSD, Depression, and Anxiety) that you use in your program are collected and how frequently they are collected.

1. *How are the data collected (e.g., paper, phone, electronic)?*
2. **How frequently are the data collected for each patient? (e.g., upon admission to the program, at discharge, 3 months and 6 months post-discharge)?*
   1. **Prompt, if needed:** Are all the measures collected at the same intervals? If not, which ones differ?
3. *Who collects the data (i.e., what role)?*
4. *Who enters the data (i.e., what role) and where is the data entered (i.e., EHR, Excel)?*
5. **How does your program manage and analyze the data collected?*
   1. *What are the biggest challenges your program has encountered regarding collection and analysis of program data? (*resource needs)
   2. *What resources or assistance might be helpful for data collection and analysis?

Since our last call, you completed the Program Characteristics Worksheet and you provided the outcome measures that your program is using.

1. **How do you use these data for improving your program outcomes?*
   1. How well do these measures capture the activities and outcomes for your program?
   2. *What are your current needs for maximizing the use of your data?*
2. Has your program used Behavioral Health Labs (BHL) or BHL Touch to collect Veteran outcomes data?

**Note:** BHL is in EHR while BHL Touch is an external system like Qualtrics – patients can fill them out in their own time on their own time

- 1. **Optional Prompts:** How do you collect the BHL data? (e.g., Who collects the data? When do you collect the data?)
  2. What are the benefits of using BHL?
  3. What issues have you had using BHL?

1. Who do you share your outcomes data with outside of your program? (e.g., locally at your facility, to the VISN, to PMOP, for CARF accreditation)

**Note:** The purpose of this question would be to try and get an understanding of the QI data they are collecting. If they do not think it would be a good measure of their program, then they may resist the data being used to "score" or "rate" the program.

- 1. What about other dissemination practices of data outside of your IPRP? (e.g., potential publications, presentations, etc.)

1. *In the future, PMOP may want to develop a national data sharing process. What are your thoughts on this?

**Note:** PMOP is interested to know down the road the content information TC will provide about the program outcomes & national data sharing--sites may decide to use it for their CARF surveys to help support them in the future.

- 1. What opportunities do you think this national data sharing process might provide?
  2. What are your potential concerns (e.g., threats) about this national data sharing process?
  3. What would you anticipate will be your needs to best facilitate a national data sharing process?

**Collaboration**

During the first interview, we talked about your collaborations at your facility. Today, we would like to better understand how your program may be connected to other sites.

1. Does your program interact or collaborate with other IPRPs? If so, how?
   1. Have you encountered any challenges collaborating with other IPRPs?
2. How does your program connect to other pain management programs or resources that are outside of your facility?
   1. Does your IPRP work with Clinical Resource Hubs or other virtual programs?
   2. Does your IPRP work with other PMOP initiatives?
   3. **Optional Prompts:** Use of programs in the local community (e.g., swimming pool at YMCA) vs. programs at other VAs?

**Closing**

1. Is there anything else that you would like PMOP to know about your program?

**Program Characteristics Worksheet**

| Category | Item | | | | | | |
| --- | --- | --- | --- | --- | --- | --- | --- |
| *General* | Participant name: _______________ | | | | | | |
|  | Year the program began: __________ | | | | | | |
| *Program Time Commitment* | Length of program: ____ Weeks | | | | | | |
|  | Number of days per week enrollees participate in the program: ____ Days/Week | | | | | | |
|  | On programming days, how many hours of treatment do participants receive?  ____ Hours/Day | | | | | | |
|  | Total program treatment hours: ____ Hours | | | | | | |
| *Program Description* | Please indicate which modes of delivery are offered for a course of your IPRP program. Select all that apply. | | | | | | |
|  | Virtual/Telehealth | Yes | | | | No | |
|  | In Person | Yes | | | | No | |
|  | Hybrid | Yes | | | | No | |
|  | Please indicate which specialties are represented in your IPRP program. | | | | | | |
|  | Physician | Yes | No | | | FTE: __________ | |
|  | Nurse Practitioner | Yes | No | | | FTE: __________ | |
|  | Physician Assistant | Yes | No | | | FTE: __________ | |
|  | Psychologist | Yes | No | | | FTE: __________ | |
|  | Social Worker | Yes | No | | | FTE: __________ | |
|  | Physical Therapist | Yes | No | | | FTE: __________ | |
|  | Occupational Therapist | Yes | No | | | FTE: __________ | |
|  | Recreational Therapist | Yes | No | | | FTE: __________ | |
|  | Aqua/Hydrotherapist | Yes | No | | | FTE: __________ | |
|  | Clinical Pharmacy Practitioner | Yes | No | | | FTE: __________ | |
|  | Chaplain | Yes | No | | | FTE: __________ | |
|  | Dietician | Yes | No | | | FTE: __________ | |
|  | Registered Nurse (RN) | Yes | No | | | FTE: __________ | |
|  | Licensed Practical Nurse (LPN) | Yes | No | | | FTE: __________ | |
|  | Acupuncturist | Yes | No | | | FTE: __________ | |
|  | Chiropractor | Yes | No | | | FTE: __________ | |
|  | Whole Health Coach | Yes | No | | | FTE: __________ | |
|  | Other (please list): __________ | Yes | No | | | FTE: __________ | |
|  | Please indicate which services are provided. Select all that apply. | | | | | | |
|  | Behavioral Therapy | Yes | | | No | | |
|  | Medication Management | Yes | | | No | | |
|  | Occupational Therapy | Yes | | | No | | |
|  | Physical Therapy | Yes | | | No | | |
|  | Pain Neuroscience Education | Yes | | | No | | |
|  | Aqua/Hydrotherapy | Yes | | | No | | |
|  | Therapeutic Recreation | Yes | | | No | | |
|  | Yoga | Yes | | | No | | |
|  | Tai Chi | Yes | | | No | | |
|  | Acupuncture | Yes | | | No | | |
|  | Whole Health Coaching | Yes | | | No | | |
|  | Other (please list): __________ | Yes | | | No | | |
| *Collaboration* | Does your program share resources with any of the following programs? | | | | | | |
|  | Outpatient Pain Clinic | | | Yes | | | No |
|  | Clinical Resource Hub (CRH) | | | Yes | | | No |
|  | Active Management of Pain (AMP) | | | Yes | | | No |
|  | Primary Care – Mental Health Integration (PCMHI) | | | Yes | | | No |
|  | Mental Health Clinic | | | Yes | | | No |
|  | Whole Health | | | Yes | | | No |
|  | PTSD Clinic | | | Yes | | | No |
|  | Pain Empowerment Anywhere Program (PEAK) | | | Yes | | | No |
|  | Other (please list): __________ | | | Yes | | | No |
| *Referrals and Enrollment* | Approximate number of individuals (patients) referred to the program in the previous fiscal year (FY23)?  ____ Patients | | | | | | |
|  | Of those referred, how many individuals were accepted to the program?  ____ | | | | | | |
|  | Of those referred, how many individuals were ultimately enrolled?  ____ | | | | | | |
|  | Of the patients who enrolled, how many individuals completed the program?  ____ | | | | | | |
| *Program Outcomes* | Which of the following pain outcomes does your program collect? Select all that apply. | | | | | | |
|  | Pain Numeric Rating Scale (NRS) | | | Yes | | | No |
|  | Defense and Veterans Pain Rating Scale (DVPRS) | | | Yes | | | No |
|  | Pain, Enjoyment, and General Activities (PEG) | | | Yes | | | No |
|  | Pain Catastrophizing Scale (PCS) | | | Yes | | | No |
|  | Patient Health Questionnaire (PHQ-9) | | | Yes | | | No |
|  | Generalized Anxiety Disorder (GAD-7) | | | Yes | | | No |
|  | Other (please list): __________ | | | Yes | | | No |
